# Management of Liver Abscess in Children: Our Experience

**DOI:** 10.5005/jp-journals-10018-1206

**Published:** 2017-05-05

**Authors:** Mukta Waghmare, Hemanshi Shah, Charu Tiwari, Kiran Khedkar, Suraj Gandhi

**Affiliations:** 1 Department of Pediatric Surgery, Topiwala National Medical College & B.Y.L. Nair Charitable Hospital, Mumbai, Maharashtra, India

**Keywords:** Catheter drainage, Liver abscess, Percutaneous aspiration, Predisposing factors.

## Abstract

**Introduction:**

Liver abscess is common in pediatric population in India. Children have unique set of predisposing factors and clinical features. Liver abscesses are infectious, space-occupying lesions in the liver; the two most common abscesses being pyogenic and amebic. Its severity depends on the source of the infection and the underlying condition of the patient.

**Materials and methods:**

A total of 34 patients less than 12 years were assessed in a retrospective study from January 2012 to 2016. Patients were assessed in terms of age of presentation, etiology, bacteriology, diagnosis, and modality of treatment.

**Results:**

The mean age of presentation was 6.3 years. Average volume of abscess was 164 cc. Nine patients (26.4%) underwent percutaneous needle aspiration under ultrasound guidance with wide bore needle (18 G disposable needle). Three patients required more than two sittings of aspiration. Patients with volume more than 80 cc were treated with catheter drainage. Twenty patients (58.8%) underwent ultrasound-guided percutaneous catheter drainage. Two patients required catheter drainage for large abscess and needle aspiration for the smaller abscess.

**Conclusion:**

Antimicrobial therapy along with percutaneous drainage constitutes the mainstay of treatment, whereas open surgical drainage should be reserved for selected cases.

**How to cite this article:** Waghmare M, Shah H, Tiwari C, Khedkar K, Gandhi S. Management of Liver Abscess in Children: Our Experience. Euroasian J Hepato-Gastroenterol 2017;7(1):23-26.

## INTRODUCTION

Liver is a major organ with dual blood supply, which predisposes it to an increased risk of infection. The incidence of pyogenic liver abscess (PLA) has decreased in the developed world, but it is still common in developing countries.^1^ Two common liver abscesses are pyogenic and amebic. The PLA may be of biliary, portal, arterial, traumatic, or cryptogenic in origin.

## MATERIALS AND METHODS

In this retrospective study, 34 patients less than 12 years of age were included, and this study was carried out from January 2012 to 2016 in a tertiary care center in West India. Patients were analyzed in terms of age, sex, presenting symptoms, and predisposing factors. Diagnostic workup included hemogram, liver function tests, coagulation profile, and ultrasonogram findings (site, number, and volume of liver abscess). Treatment modality included intravenous antibiotics and ultrasound-guided needle aspiration or ultrasound-guided pigtail catheter drainage. The resolution of the abscess was monitored by serial ultrasound examination. All patients were discharged on oral metronidazole and regular follow-up.

## RESULTS

The mean age of presentation was 6.3 years, with age range being 1.5 to 12 years. Around 16 patients (47.01%) were less than 5 years of age. There were 19 girls (55.8%) and 15 boys (44.1%). Most patients (64.7%) presented during monsoon and postmonsoon season between June and November.

Two patients were diagnosed with enteric fever 15 to 20 days prior to presentation. One patient had a preceding history of blunt trauma to the abdomen. Two patients had associated pleural effusion. No significant predisposing factors were found in the rest (n = 30). Fever and abdominal pain were the most common presenting complaints (33 patients). All patients had right hypochondriac tenderness. None of patients had icterus. Total leukocyte count was raised in 25 patients. Liver function tests including coagulation profile were normal in all patients. Diagnosis was confirmed on ultrasound and patients were started on intravenous broad spectrum antibiotics (piperacillin + tazobactam and metronidazole).

On ultrasound, the abscesses were localized in the right lobe in 25 patients (73.5%) and in left lobe in 6 (17.6%); 3 patients had abscesses involving both lobes. Single lesion was found in 26 patients (79.4%), 8 patients had two or more abscesses.

**Graph 1:**
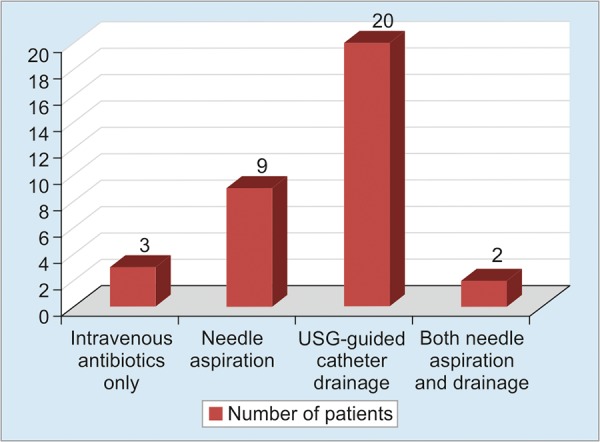
Management modalities of 34 patients with liver abscesses

Depending upon location, size, volume, and state of liquefaction of abscess, treatment modality was planned (Graph 1). Average volume of abscess was 164 cc. Three patients with small abscess not amenable to aspiration were managed with intravenous antibiotics only. Nine patients (26.4%) underwent percutaneous needle aspiration under ultrasound guidance with wide bore needle (18 G disposable needle). Three patients required more than two sittings of aspiration. Patients with volume more than 80 cc were treated with catheter drainage; 20 patients (58.8%) underwent ultrasound-guided percutaneous catheter drainage. Two patients required catheter drainage for large abscess and needle aspiration for smaller abscess.

Pus culture was negative in 26 patients (76.47%). *Staphylococcus aureus* was grown in three patients, and methicillin-resistant *S. aureus, Pseudomonas,* and *Acineto-bacter* species were grown in one patient each.

Repeat ultrasound was done when drain output decreased to less than 10 cc per day or if there was no clinical improvement. Mean duration of drain was 7.7 days (4-16 days). Patients were discharged on oral antibiotics for a duration of 3 weeks. All patients were asymptomatic on follow-up and ultrasound examination was normal.

## DISCUSSION

Pyogenic liver abscess constitutes the majority of cases, followed by amebic and fungal. Pyogenic liver abscess constitutes the majority (80%) of hepatic abscesses in children.^2-4^

The incidence of PLA has been reported to be more than 79 per 100,000 pediatric admissions in India.^5^ Amebic liver abscess is rare in children^6^ and mostly endemic in Thailand, India, Egypt, and South Africa.^7^ Amebic liver abscess develops in less than 1% of patients infected with *Entamoeba histolytica.^8^*

The predisposing factors are perforated appendicitis, chronic granulomatous disease, sickle cell disease, immunocompromised status due to malignancy, postchemotherapy, and chronic malnutrition. When the biliary tract is the source of liver abscess, there are multiple abscesses. Protein calorie malnutrition also predisposes to liver abscess in children probably due to immunosuppressed state.^1^ Kumar et al^5^ reported moderate to severe malnutrition in 27.8% patients with liver abscess. Hepatic trauma may cause localized hepatic necrosis, intrahepatic hemorrhage, and bile leakage, thus providing a suitable environment for bacterial growth.

The other causes in immunocompetent patients are intestinal infection, chronic cholangitis, umbilical vein catheterization (in neonates), and systemic bacteremia of any cause.

Approximately two-third of liver abscesses occur in the right lobe of the liver and the majorities are solitary.^1,5^ The predilection for the right hepatic lobe can be attributed to the volume of the right portal vein flow and also that the right portal vein continues in the direction of the common portal vein, while the left portal vein takes a more horizontal direction. Multiple liver abscesses constitute 20 to 25% of all cases.^9^

Most of these patients are less than 5 years of age. Clinical signs and symptoms of liver abscess are usually nonspecific with variable duration like fever, abdominal pain, and loss of appetite and nausea, which often delays the diagnosis. Hepatomegaly is usually associated with right upper quadrant tenderness.

*Staphylococcus aureus* is the most common etiological agent for PLA. Other bacteria include *Escherichia coli, Klebsiella, Enterobacter, Pseudomonas,* and sometimes anaerobes. Anaerobes constitute an important proportion of up to 30% of organisms and include microaero-philic *Streptococci.^9^* Recurrent pyogenic cholangitis may be due to *Salmonella typhi.^10^* Kumar et al^11^ have reported liver abscess as an unusual complication of enteric fever in the pediatric age group. Fungal hepatic micro-abscesses either alone or in association with splenic microabscesses may occur in children with leukemia.^12^

A large number of cases have been found without any apparent cause and have been labeled as cryptogenic.^13^ As high as 33 to 35% cases of cryptogenic liver abscess have been reported by Donovan et al^14^ and Bari et al.^13^ The abscess may also be sterile because the patient has received prior antibiotic therapy. In our study, low level of positive cultures could partly be due to prior antibiotic therapy received by patients before admission as most of patients were referred from small peripheral health centers. Blood investigations may show leukocytosis, anemia, and altered liver function tests.

**Fig. 1:**
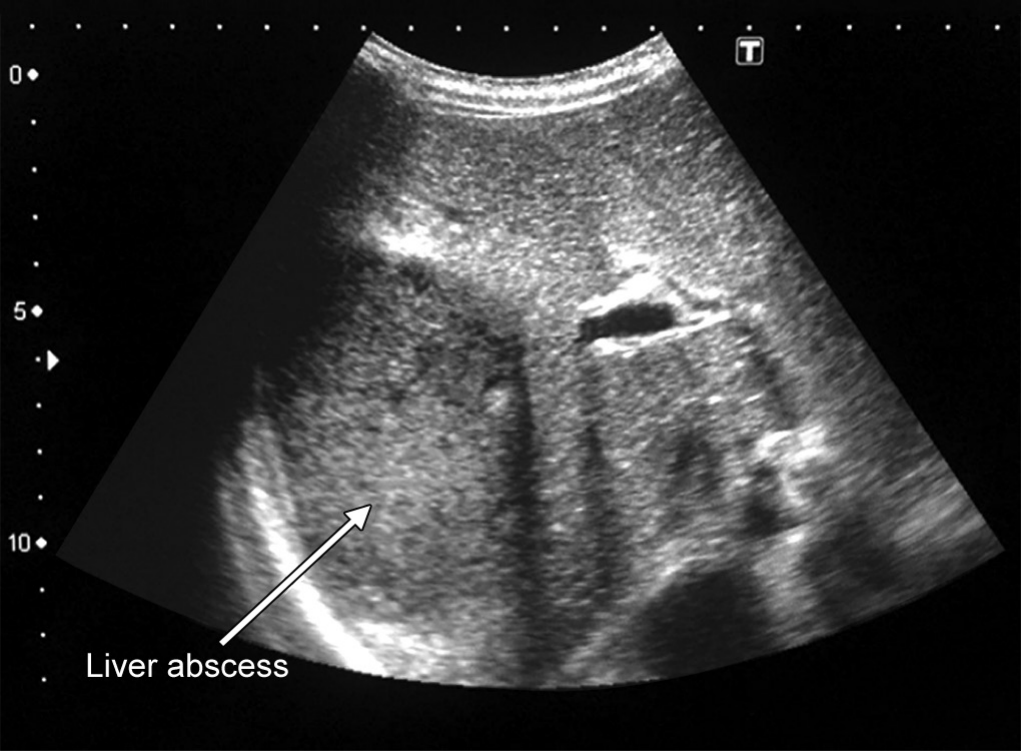
Ultrasonography showing abscess in liver

Ultrasonography of abdomen serves initial investigation to assess the site, size, and number of liver abscess (Fig. 1). Contrast-enhanced computed tomography (CT) is more sensitive in detecting even small abscesses anywhere in the liver. Liver abscesses on magnetic resonance imaging appear hypointense on T1-weighted and hyperin-tense in T2-weighted sequences. On gadolinium-enhanced sequences, there is early and continued enhancement of wall, which persists on delayed images.

Initial treatment of PLA is broad spectrum antibiotics, which cover gram positive, gram negative, and anaerobic organisms. A course of 6 weeks antibiotic therapy alone, including 2 weeks intravenously, followed by 4 weeks orally is recommended, when multiple abscesses are too small (less than 2 cm) to be drained percutaneously.

Aspiration can be attempted in solitary, unilocular lesions and on carefully selected patients.

Percutaneous drainage (PD) has now come to a cen-terstage in management of liver abscesses that require more than just a medical management. Percutaneous aspiration in conjunction with antibiotics has been recommended for unilocular liver abscess.^13,14^

Safety and efficacy of percutaneous abscess drainage in selected patients is now well established.^15^ Even multiloculated liver abscesses can be managed with aggressive percutaneous techniques that include disruption of loculations and placement of large bore sump catheters.^15^ Percutaneous drainage is indicated when there is a large volume abscess and there is risk of spontaneous rupture (specially left lobe abscesses).^15^ When there is lack of response to medical therapy with clinical signs of persistent sepsis or enlarging abscesses, or persistent symptoms.^9^

A review of various studies shows that the overall failure rate of PD ranges from 5 to 28%.^16-18^ Herman et al^19^ analyzed 48 patients with PLA and found that the failure rate of PD was 30.8%, whereas the failure rate of open surgical drainage was only 8.5%. Percutaneous management failed in patients with thick-walled abscess or containing viscid pus and in the presence of loculations. Percutaneous drainage is not indicated in the presence of ascites or when liver abscess is close to the pleura.

The indications of open laparotomy include nonre-sponse to PD together with antibiotic therapy, or when the pus is thick, multiloculated abscess or rupture into peritoneal cavity.

Facility of prompt diagnosis with imaging, PD, and better antibiotics has remarkably improved survival in last three decades. With modern management, mortality is less than 15%.^9^

## CONCLUSION

In conclusion, liver abscess in children is still very common in developing countries; PLA is more common than amebic, fungal, or other etiologies. Imaging with ultrasonography and/or CT is diagnostic. Antimicrobial therapy along with PD constitutes the mainstay of treatment, whereas open surgical drainage should be reserved for selected cases.
